# Supplementary material for: Pyruvate Dehydrogenase Kinase 1 inhibition mediated oxidative phosphorylation enhancement in cartilage promotes osteoarthritis progression
Source: BMC Musculoskelet Disord. 2023 Jul 20;24:597. doi: 10.1186/s12891-023-06585-6 (PMC10357736; doi:10.1186/s12891-023-06585-6)
Supplement: Supplementary file 1 — Additional file 1. [file 12891_2023_6585_MOESM1_ESM.zip › KEGG.Gsea.1653622432464/KEGG_ASTHMA.html]

Details for gene set KEGG\_ASTHMA[GSEA]

|  || Dataset | 55457\_collapsed\_to\_symbols.cls.cls #OA\_versus\_normal.cls.cls #OA\_versus\_normal\_repos |
| Phenotype | cls.cls#OA\_versus\_normal\_repos |
| Upregulated in class | OA |
| GeneSet | KEGG\_ASTHMA |
| Enrichment Score (ES) | 0.24681069 |
| Normalized Enrichment Score (NES) | 0.87604094 |
| Nominal p-value | 0.6507937 |
| FDR q-value | 0.86820894 |
| FWER p-Value | 1.0 |
Table: GSEA Results Summary

  

Fig 1: Enrichment plot: KEGG\_ASTHMA      
 Profile of the Running ES Score & Positions of GeneSet Members on the Rank Ordered List

  

| SYMBOL | TITLE | RANK IN GENE LIST | RANK METRIC SCORE | RUNNING ES | CORE ENRICHMENT || 1 | HLA-DMB | "major histocompatibility complex, class II, DM beta [Source:HGNC Symbol;Acc:HGNC:4935]" | 679 | 0.598 | 0.0283 | Yes |
| 2 | MS4A2 | membrane spanning 4-domains A2 [Source:HGNC Symbol;Acc:HGNC:7316] | 1203 | 0.475 | 0.0521 | Yes |
| 3 | HLA-DQA1 | "major histocompatibility complex, class II, DQ alpha 1 [Source:HGNC Symbol;Acc:HGNC:4942]" | 1334 | 0.452 | 0.1033 | Yes |
| 4 | HLA-DMA | "major histocompatibility complex, class II, DM alpha [Source:HGNC Symbol;Acc:HGNC:4934]" | 1769 | 0.388 | 0.1222 | Yes |
| 5 | TNF | tumor necrosis factor [Source:HGNC Symbol;Acc:HGNC:11892] | 2098 | 0.350 | 0.1442 | Yes |
| 6 | HLA-DOB | "major histocompatibility complex, class II, DO beta [Source:HGNC Symbol;Acc:HGNC:4937]" | 2147 | 0.344 | 0.1871 | Yes |
| 7 | FCER1A | Fc fragment of IgE receptor Ia [Source:HGNC Symbol;Acc:HGNC:3609] | 2564 | 0.295 | 0.1948 | Yes |
| 8 | FCER1G | Fc fragment of IgE receptor Ig [Source:HGNC Symbol;Acc:HGNC:3611] | 2752 | 0.275 | 0.2175 | Yes |
| 9 | HLA-DQB1 | "major histocompatibility complex, class II, DQ beta 1 [Source:HGNC Symbol;Acc:HGNC:4944]" | 2840 | 0.266 | 0.2468 | Yes |
| 10 | HLA-DOA | "major histocompatibility complex, class II, DO alpha [Source:HGNC Symbol;Acc:HGNC:4936]" | 4344 | 0.125 | 0.1469 | No |
| 11 | IL5 | interleukin 5 [Source:HGNC Symbol;Acc:HGNC:6016] | 5277 | 0.050 | 0.0813 | No |
| 12 | RNASE3 | ribonuclease A family member 3 [Source:HGNC Symbol;Acc:HGNC:10046] | 5463 | 0.036 | 0.0718 | No |
| 13 | HLA-DPB1 | "major histocompatibility complex, class II, DP beta 1 [Source:HGNC Symbol;Acc:HGNC:4940]" | 5486 | 0.033 | 0.0746 | No |
| 14 | CD40LG | CD40 ligand [Source:HGNC Symbol;Acc:HGNC:11935] | 5529 | 0.030 | 0.0754 | No |
| 15 | HLA-DRA | "major histocompatibility complex, class II, DR alpha [Source:HGNC Symbol;Acc:HGNC:4947]" | 6550 | -0.046 | 0.0024 | No |
| 16 | IL4 | interleukin 4 [Source:HGNC Symbol;Acc:HGNC:6014] | 6673 | -0.054 | 0.0002 | No |
| 17 | CD40 | CD40 molecule [Source:HGNC Symbol;Acc:HGNC:11919] | 6825 | -0.065 | -0.0028 | No |
| 18 | CCL11 | C-C motif chemokine ligand 11 [Source:HGNC Symbol;Acc:HGNC:10610] | 6907 | -0.073 | 0.0008 | No |
| 19 | HLA-DRB1 | "major histocompatibility complex, class II, DR beta 1 [Source:HGNC Symbol;Acc:HGNC:4948]" | 7087 | -0.086 | -0.0014 | No |
| 20 | IL10 | interleukin 10 [Source:HGNC Symbol;Acc:HGNC:5962] | 8855 | -0.221 | -0.1086 | No |
| 21 | PRG2 | "proteoglycan 2, pro eosinophil major basic protein [Source:HGNC Symbol;Acc:HGNC:9362]" | 9135 | -0.242 | -0.0975 | No |
| 22 | HLA-DPA1 | "major histocompatibility complex, class II, DP alpha 1 [Source:HGNC Symbol;Acc:HGNC:4938]" | 9481 | -0.273 | -0.0873 | No |
| 23 | IL3 | interleukin 3 [Source:HGNC Symbol;Acc:HGNC:6011] | 9850 | -0.306 | -0.0744 | No |
| 24 | EPX | eosinophil peroxidase [Source:HGNC Symbol;Acc:HGNC:3423] | 10492 | -0.372 | -0.0738 | No |
| 25 | IL9 | interleukin 9 [Source:HGNC Symbol;Acc:HGNC:6029] | 11309 | -0.472 | -0.0732 | No |
| 26 | IL13 | interleukin 13 [Source:HGNC Symbol;Acc:HGNC:5973] | 12173 | -0.636 | -0.0540 | No |
| 27 | HLA-DRB4 | "major histocompatibility complex, class II, DR beta 4 [Source:HGNC Symbol;Acc:HGNC:4952]" | 12636 | -0.815 | 0.0205 | No |
Table: GSEA details [plain text format]

  

Fig 2: KEGG\_ASTHMA      
 Blue-Pink O' Gram in the Space of the Analyzed GeneSet

  

Fig 3: KEGG\_ASTHMA: Random ES distribution      
 Gene set null distribution of ES for **KEGG\_ASTHMA**
